# Plaster as an Impression Material

**Published:** 1869-05

**Authors:** Reuben K. George


### ARTICLE III.

### *Plaster as an Impression Material.*

By Reuben K. George, D.D-S.

Plaster as an impression material is one of the most important, if not the most important, of all the minerals made use of in mechanical dentistry.

There are three other substances used for taking impressions, Wax of the animal kingdom, Gutta-Percha of the vegetable, and Paraffine of the fossil mineral kingdom. But plaster comes first on the list, and is of the mineral kingdom, being known in chemistry as sulphate of lime, (Symbols $Ca\ OSO_3$ or $Ca\ SO_4+2H_2O$ by the new notation,) more properly the sulphate of the oxide of calcium, because it first forms with sulphuric acid. It is known in dentistry as plaster or calcined plaster; to the agriculturist as plaster of Paris, from its having been originally found in large quantities, at Monmartre, in the environs of Paris. It is known in commerce as Gypsum, and consists of twenty-eight parts of lime, forty parts of sulphuric acid and eighteen parts of water. It is found in large quantities in France, Nova Scotia and some parts of the United States. It is very often associated with rock salt, and also exists in four forms, Selenite, (which is a crystalline variety) Amorphous, Alabaster, and Fibrous. The amorphous is the variety prepared for the use of the dental profession.

Sulphate of lime is soluble in five hundred parts of cold water, and its solubility is slightly increased by heating the mixture; it is also more soluble in water containing chloride of ammonia or nitrate of potassa.

Plaster is also a useful agent in the dental laboratory for many other purposes besides taking impressions.

In preparing it for the use of the dental laboratory, the hardest pieces should be chosen and ground to powder, not too fine, however, for it is desirable to have some preparations of it fine and others somewhat coarse.

It then put into a large iron kettle and raised to a temperature of from 260°F. to 300°F. at which temperature it boils very much like water; just before the ebullition ceases the heat should be removed, and it then becomes what is known as calcined plaster. By this process of boiling or calcining, the water of crystallization is driven off to some extent, but it should not be heated higher than 300° or all the water of crystallization may be driven off, which converts it into an anhydrate which cannot be used in the laboratory because its setting properties are lost; the very properties which make it so applicable to dentistry. Another process for preparing it, is to roast it in the lump in a furnace heated to 300° and then reduce it to powder. On the addition of water to the plaster it forms a batter which converts it into the same hard substance as before, by giving back its equivalents of water; it is then a hydrate.

Plaster has been in use as an impression material for full upper dentures for a great many years, and it has almost wholly superceded wax in all full cases, and is undoubtedly the best material which can be used. It is now coming into very general use as a material for taking impressions for partial sets of teeth, as it can be used in the most complicated cases in connection with the gutta-percha mouth cup.

It possesses the property of taking a perfect impress of all the soft parts in their normal conditions and relations to each other, and from its great plasticity, it copies the most minute lines of the softest tissues, with perfect accuracy; a property which no other material possesses to the same degree. Another great advantage is the hardness which it attains in a short time and before it is taken from the mouth. One advantage which it possesses over wax and its compounds, or gutta-percha and its compounds, is that of being much more plastic when applied, therefore requiring very little pressure; it also becomes much harder in the same length of time. The advantage resulting from the slight pressure it requires is that parts are not displaced. Besides plaster is much more yielding when in a plastic state and much harder when it has set or when its affinity for water has been satisfied, than wax or gutta-percha.

In grinding plaster we should be careful not to grind too fine; and to grind too coarse would be equally objectionable. A proper way to judge of this is by rubbing the powdered plaster between the fingers.

In using fine or coarse qualities of plaster all the requirements of a good impression are not fulfilled: In the first place if the plaster is too fine, our impression will be too soft, and secondly, if the plaster is too coarse our impression will expand after it has been taken from the mouth, which will destroy more or less the relations of the parts to each other. The reason why the coarse plaster expands is, that the particles do not become thoroughly saturated with water until after the impression has been taken from the mouth; and afterwards absorbing their full supply of water, they are thereby more or less expanded. We should therefore avoid having our plaster too fine or too coarse, and should supply ourselves with two grades of this material for impressions.

In taking impressions where we intend to use the vulcanite base, it is advisable to use rather fine plaster, for the reason that the vulcanite plate will be an exact counterpart of the impression or model without any shrinkage or expansion. But for swaged work we desire a slight expansion of the impression, so that our model will be slightly larger than the parts of the mouth really are, and our reason for this is, that the contraction of the metal plate will just about counterbalance the expansion of the impression, hence the great advantage plaster has over all other impression materials.

Some of the other impression materials have a contractile power, but none possess the expansive power of plaster; which is a very essential property when we are constructing swaged work or teeth on metal base.

In taking an impression with plaster, we are not so liable to spoil it in withdrawing the cup from the mouth, nor are we so liable to get a rocking impression of the mouth, that is one which will cause the plate to press too hard on the roof of the mouth. When the plaster begins to harden there is not so much danger of its becoming displaced, as it rather adheres to the mucous membrane of the mouth. I contend therefore, that it is a great deal easier to take an impression with plaster than with any other material, for the accidents liable to occur during this process are very greatly reduced in favor of plaster. After the plaster has set we have an accurate impression, and there is no liability of destroying it by giving it a rocking motion in taking it from the mouth. This liability of destroying the accuracy of the impression in loosening and withdrawing it from the mouth, is open to all other materials for impressions more or less. Again a plaster impression when it has become hard, is not liable to disarrangement in removing it from the mouth, for we may break away some delicate parts, or, where there are undercuts, may disengage pieces, but these will all be broken with a sharp fracture, and can be placed in their exact positions, without destroying the integrity of the impression in the least degree. Besides there is no danger of the dragging behind which is so great an objection to wax. In other words plaster possesses most of the requirements for a perfect impression material. If we were confined to any one material we shonld greatly prefer plaster, for wax and gutta-percha possess some advantages over plaster in special cases, where the parts of the mouth possess different degrees of hardness, yet in all those cases where the parts of the mouth are either uniformly soft or uniformly hard, plaster is to be much preferred. Now in cases where a part of the alveolus is soft and the rest hard, or where there are loose folds of mucous membrane, or where there is a soft ridge and a hard palate, or a hard ridge and a soft palate, wax or gutta-percha, possess advantages over plaster. For in such cases we wish to press up the soft or loose parts, so as to condense them to some extent, and hence the necessity for the use of wax or gutta-percha or some of their compounds. Now in choosing and buying plaster we are very likely to be deceived, for there is a great difference in the varieties met with as regards their setting properties, and it requires some experience to select the good from the bad. There are several simple methods however, by which we may judge of the quality of plaster. One way is to take up a handful and close the fingers upon it; if it adheres together like flour it may be considered a very good article, though it is not always best to depend upon this test. The surest test is to mix some of the plaster with a little water and time the setting by the watch. Plaster which will set in from three to ten minutes and become hard, may be considered as first class. The hardening of plaster may be divided into two stages; the first extends from the time the water is applied until it is too hard to take an accurate impress; the second extends from this time until it is solid. Now we may change these stages into long or short, by certain precautions in mixing; if we mix the plaster very thin it will be much longer in passing through the first stage, but if we mix it thick at first, it is longer passing through the second stage. Now this difference in the time of setting is of great practical importance to us in taking impressions; for in preparing the plaster and putting it into the impression cup and introducing it into the mouth, we require the most time, and this of course reduces the time it is necessary for it to remain in the mouth, to become solid. Thus it is very nicely adapted for impressions in this particular.

In mixing plaster that will set in ten minutes, we may lengthen the first stage two minutes, By mixing it thin the first stage will require six minutes and the second stage four minutes, or *vice versa*. We also have it in our power to hasten or retard the setting properties of a given plaster, by mixing with it certain agents. For hastening the setting we add a small quantity of chloride of sodium or sulphate of potash, or we may make a weak solution of either of these agents and use them for mixing up the plaster. For retarding the setting we may use a mixture of dilute white glue, sugar diluted, molasses, water or beer. Calcined plaster should be kept in tightly closed tin cans, so as to keep the air from it, for if it is exposed to the air it will absorb moisture, which satisfies its great affinity for water, and consequently its setting property will be much deteriorated. The best time to place the plaster in the cup for taking an impression is just when it is stiff enough to build up, then when you can turn the cup bottom upwards and the plaster does not drop off, it may be inserted into the mouth; however, it may still be considered in a good condition for taking an impression so long as it leaves a glassy surface after passing the spatula over it; after this it is too hard, for on applying pressure it will crack, and press apart.

In mixing plaster, we may prevent the presence of air bubbles by first putting the water in the bowl and then by degrees sprinkle the plaster in from the blade of the spatula until nearly all the water is absorbed. When stirring we should be careful not to raise the spatula above the surface so as to mix air with it, as air bubbles on the surface of the impression will destroy its accuracy. In using plaster we should also avoid letting it remain in the mouth too long, for after it has set it will absorb moisture from the mucous membrane of the mouth and will adhere very closely, causing considerable difficulty in loosening it, and much pain to the patient; sometimes the soft parts of the mouth are torn, or otherwise injured. We can always determine the exact time it will take a certain quality of plaster to set by experimenting and timing the setting, or by trying what is left in the bowl. When the plaster breaks with a sharp fracture it is hard enough to withdraw; but we should always make some allowance on account of the warmth of the mouth, which causes the plaster in the impression cup to set quicker than that which remains in the bowl.

---

